# Switch of noninvasive ventilation (NIV) to continuous positive airway pressure (CPAP) in patients with obesity hypoventilation syndrome: a pilot study

**DOI:** 10.1186/s12890-017-0391-9

**Published:** 2017-03-14

**Authors:** Sarah Orfanos, Dany Jaffuel, Christophe Perrin, Nicolas Molinari, Pascal Chanez, Alain Palot

**Affiliations:** 1 Aix-Marseille University, Faculté de médecine, 27 Boulevard Jean Moulin, 13005 Marseille, France; 2 Département de Pneumologie, Hôpital Arnaud de Villeneuve, CHRU de Montpellier, 371 Avenue du Doyen Giraud, 34295 Montpellier, Cedex 5 France; 3 Service de Pneumologie, Pôle des Spécialités Médicales, Centre Hospitalier de Cannes, 15 avenue des Broussailles, 06401 Cannes, France; 4 INSERM U1046, Université de Montpellier 1 et Département Médical d’Information, Centre Hospitalier Universitaire, 34090 Montpellier, France; 5 Clinique des Bronches, Allergies et du Sommeil, Assistance Publique Hôpitaux de Marseille, France et INSERM U1067, CNRS UMR 7333 Aix Marseille Université, 13015 Marseille, France

**Keywords:** Obesity hypoventilation syndrome, Non invasive ventilation, Continuous positive airway pressure, Bilevel positive airway pressure, Obstructive sleep apnea

## Abstract

**Background:**

Obesity is a major worldwide public health issue. The main respiratory complication stemming from obesity is obesity hypoventilation syndrome (OHS). Most of the OHS patients diagnosed during an exacerbation are treated with non invasive ventilation (NIV). Up to date, no prospective study has demonstrated in real life conditions the feasibility of a systematic protocoled switch of NIV to continuous positive airway pressure (CPAP), once stability is achieved.

**Methods:**

In this prospective study, we included stable patients with OHS, with moderate to severe concomitant obstructive sleep apnea (OSA) and without obstructive pulmonary disease, who had been undergoing NIV for more than 2 months. The following measurements were performed, first with NIV and then after the switch to CPAP: diurnal arterial blood gas measurements; nocturnal oximetry and capnometry; mean compliance and AHI; measures of quality of life and quality of sleep.

**Results:**

22/30 patients accepted to participate in the study and 15/22 patients completed the study. There were no significant differences for pooled data in diurnal alveolar blood gases, nocturnal capnometry (*p* = 0.534), nocturnal oximetry (*p* = 0.218), mean compliance (*p* = 0.766), mean AHI (*p* = 0.334), quality of life or quality of sleep. Eighty percent of the patients treated in this study favored CPAP over NIV.

**Conclusion:**

This pilot study showed in real life conditions the possibility of a systematic switch of NIV to CPAP, in most stable patients with OHS, with similar efficacy on diurnal and nocturnal alveolar gas exchange, quality of life and quality of sleep.

**Trial registration:**

ISRCTN13981084. Registered: 27 February 2017 (retrospectively registered)

## Background

Obesity is a major worldwide public health issue [1]. The main respiratory complication stemming from obesity is obesity hypoventilation syndrome (OHS). OHS was defined, in 1999, by the American Academy of Sleep Medicine as the association of obesity (BMI ≥ 30 kg/m^2^); daytime hypercapnia (paCO2 > 45 mm Hg); and sleep disordered breathing, after excluding other causes of hypoventilation [2]. OHS is a frequent condition with the prevalence of OHS in patients with a BMI ≥ 35 kg/m^2^ being 31% [3].

OHS is an underdiagnosed and undertreated condition [3, 4], despite the fact that a treatment with positive airway pressure improves quality of life and decreases morbidity in these patients [3, 5]. The application of a positive airway pressure through noninvasive ventilation (NIV) or continuous positive airway pressure (CPAP), decreases mortality in patients presenting with OHS [4, 6, 7].

To date, the main unresolved question is to determine the respective position of NIV and CPAP when treating these patients. If some studies have attempted to determine different OHS’s phenotypes to predict the outcome of CPAP therapy [8–10]; only two studies prospectively compared NIV and CPAP treatment in OHS [5, 11]. To date, the superiority of NIV over CPAP in the treatment of OHS has not been proven. However, 26 to 50% of patients suffering from OHS are diagnosed in an acute setting [7, 8, 11, 12], and most often treated with NIV which remains their usual ventilatory mode for the rest of their life.

Once stability has been reached, the question then arises as to whether NIV should be switched to CPAP. To our knowledge, no prospective trial has evaluated in real life conditions the feasibility of a protocol switching stable patients from NIV to CPAP.

The purpose of this study was to test the feasibility and effect of a standardized protocol, switching patients with OHS, from NIV to CPAP. We hypothesized that there will be no difference in efficacy after switching NIV to CPAP, on AHI, diurnal and nocturnal alveolar gas exchange (daytime arterial blood gas (ABG), nighttime transcutaneous oxygen saturation and transcutaneous measurement of pCO2 (ptCO2)), but also on sleepiness, quality of sleep and quality of life (Epworth Sleepiness Scale (EPS), Pittsburgh Sleep Quality Index (PSQI), Severe Respiratory Insufficiency questionnaire (SRI)).

## Methods

### Patients

Patients over 18 years old, suffering from OHS, defined as an association of obesity (BMI ≥ 30 kg/m^2^) and daytime hypercapnia (paCO2 > 45 mm Hg), who had been undergoing NIV for more than 2 months, were prospectively recruited from February 2015 to February 2016 in the pulmonology wards of the university hospital (hôpital Nord) and the military hospital (hôpital Laveran) in Marseille, France. To be included, patients had to be clinically stable for at least 4 weeks before enrollment in the study (no hospitalization or emergency admission). The exclusion criteria were: patients who presented hypercapnia secondary to other causes (obstructive pulmonary diseases (FEV1/FVC < 70), interstitial lung diseases, neuromuscular or chest wall diseases, severe hypothyroidism or congenital central hypoventilation syndrome), and patients unable to give informed consent.

This trial respected the ethical principles of the declaration of Helsinki and was approved by the local ethic committee of the Assistance Publique Hôpitaux de Marseille (AP-HM). Written informed consent was obtained from all patients. The use of data was approved by the Data Privacy Officer of the hospitals (AP-HM).

### Study protocol

Patients who had been undergoing NIV for at least the last 2 month, were prospectively included in the study by the investigators, during a follow-up outpatient clinic.

During this clinic, the following clinical information were collected: history of the diagnosis of OHS, occurrence of acute exacerbations, NIV settings: brand, mode, respiratory rate (RR), expiratory positive airway pressure (EPAP), inspiratory positive airway pressure (IPAP) and interfaces.

The settings were in accordance with the American Academy of Sleep Medicine (AASM) recommendations [13]. NIV settings were optimized in order to avoid patient-ventilator dyssynchrony and to correct remaining respiratory events during sleep, according to the recommendations of the somnoNIV group [14].

Quality of life was evaluated using the SRI questionnaire; daytime sleepiness was evaluated using the ESS questionnaire; and quality of sleep was evaluated using the PSQI questionnaire.

Finally, an ABG analysis was performed, during the consultation, between 10 and 12 am in an awake, seated patient, at rest for at least 10 min.

During one night with NIV, at home, the patient underwent: (i) nocturnal oximetry using a pulse oximeter (Rescan® software, Resmed®, Australia, New South Wales, Bella-Vista), on which were calculated the percentage of recording time with SaO2 < 90% (RT < 90%) and the oxygen desaturation index (ODI) (defined as the number of time per hour the oxygen saturation drops by ≥ 4%); (ii) nocturnal transcutaneous capnometry using a transcutaneous capnometer (Tosca 500®, Radiometer®, Crawley, UK), on which was calculated the mean ptCO2; (iii) a report of the mean compliance, the median leak, the mean tidal volume and the mean AHI index of the last month spent with NIV was extracted (Rescan® software for Resmed®, Australia New South Wales, Bella-Vista NIV and Directview® for Philips-Respironics®, Murrysville, Pennsylvania, USA NIV).

After undergoing these procedures, the patients underwent NIV withdrawal for seven nights. At the end of this week, pulmonary function tests (PFT) were performed according to the American Thoracic Society and European Respiratory Society 2005 guidelines [15], and an overnight polygraphy during spontaneous breathing was performed (Alice PDx®, Philips-Respironics®, Murrysville, Pennsylvania). Polygraphic recordings were analyzed by a blinded pulmonologist certified in sleep medicine, respiratory events were scored manually according to the AASM 2012 manual [16]. The AHI, RT < 90% and ODI on this polygraphy during spontaneous breathing were reported. At this stage of the trial, patients exhibiting obstructive ventilatory disorder (FEV1/FVC < 70) or mild OSA (AHI < 15/h), were not switched to CPAP and continued their treatment with NIV. All the other patients were switched to CPAP, the settings were as follow: constant pressure, EPAP 2 cm H_2_O above the EPAP of the previous NIV to compensate for the absence of an IPAP on the CPAP, settings were not adjusted until measurements were repeated more than a month after this switch.

**Fig. 1:**
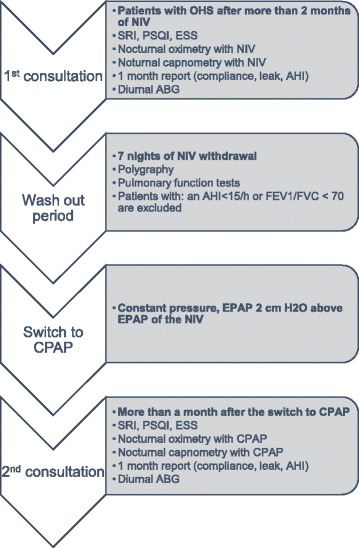
Study protocol. Definition of abbreviations: OHS: Obesity Hypoventilation Syndrome; NIV: Non Invasive Ventilation; SRI: Severe Respiratory Insufficiency questionnaire; PSQI: Pittsburgh Sleep Quality Index; ESS: Epworth Sleepiness Scale; AHI: Apnea Hypopnea Index; ABG: Arterial Blood Gas; FEV1: Forced Expiratory Volume in 1 s; FVC: Forced Vital Capacity; EPAP: Expiratory Positive Airway Pressure; CPAP: Continuous Positive Airway Pressure

After more than a month with CPAP; SRI, ESS and PSQI questionnaires were reevaluated. An ABG analysis was performed in the same conditions as previously described. During one night with CPAP, at home, the patient underwent: (i) nocturnal oximetry; (ii) nocturnal transcutaneous capnometry; (iii) a report of the mean compliance, the median leak and the mean AHI index of the last month spent with CPAP was extracted. (Fig. 1).

As a secondary analysis, we analyzed the characteristics of patients failing NIV and CPAP using Salord et al. criteria to define failure: more than 30% of recording time spent below 90% saturation or paCO2 > 45 mm Hg, to determine if a characteristic could be used as a prognostic factor of ventilation failure [10]﻿.

### Statistical analysis

Continuous data were expressed in means and SDs and categorical data were expressed as percentages and absolute.

Normality was assessed using Shapiro-Wilk normality test. Intra-group changes in variables measured at first with NIV and after the switch to CPAP were assessed using paired t-tests or non-parametric Wilcoxon tests depending on normality.

When comparing two independent groups (NIV failure, NIV success), unpaired t-tests or non-parametric Mann–Whitney tests depending on normality were used for continuous variables and x^2^ tests for categorical variables.

## Results

### Study population

**Fig. 2:**
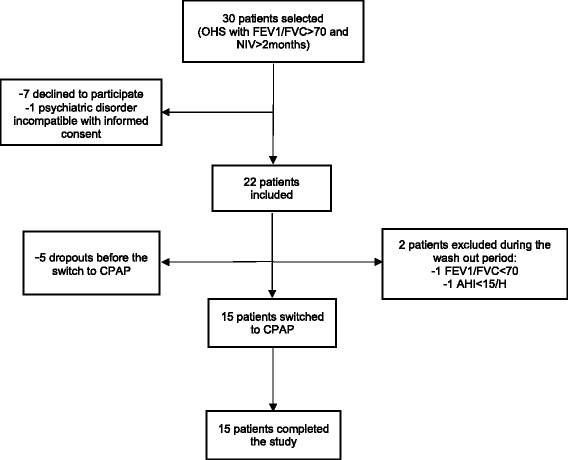
Flow chart. Definition of abbreviations: OHS: Obesity Hypoventilation Syndrome; FEV1: Forced Expiratory Volume in 1 s; FVC: Forced Vital Capacity; NIV: Non Invasive Ventilation; CPAP: Continuous Positive Airway Pressure; AHI: Apnea Hypopnea Index

Twenty-two patients were included. Five dropped-out due to unwillingness to be hospitalized for the polygraphy prior to the switch to CPAP. Two patients were excluded prior to the switch, one had an obstructive syndrome on the PFT, and the other had an AHI < 15/h on the polygraphy. Fifteen of the 30 patients who were initially selected, were switched to CPAP (Fig. 2).

Anthropometric characteristics of the patients are shown in Table 1.

**Table 1:** Baseline characteristics

Baseline characteristics	*n* = 15
Sex (male)	5 (33.3%)
Age (years)	68.8 ± 11.2
BMI (kg/m^2^)	41.7 ± 7.16
Neck circumference (cm)	43.6 ± 4.5
Waist/Hip ratio	0.95 ± 0.08
pH on ABG before initiating NIV	7.38 ± 0.06
paCO2 on ABG before initiating NIV (mmHg)	57.7 ± 7.6
paO2 on ABG before initiating NIV (mmHg)	62.6 ± 18.8
HCO3- before initiating NIV (mmol/L)	31.8 ± 5.1
Actual or past smokers	7 (46.7%)
Pack per year	38.1 ± 30.9
Cardiovascular comorbidities	13 (86.7%)
Hypertension	12 (80%)
Dyslipidemia	12 (80%)
Diabetes	9 (60%)
Cardiac disease	12 (80%)
Ischemic cardiac disease	5 (33.3%)
Cardiac arrhythmia	5 (33.3%)
LVEF (%)	56 ± 14.3
Diastolic dysfunction	8 (61.5%)
Diagnosis made during an exacerbation	12 (80%)

Data are expressed in n (%) and mean ± standard deviation

Definition of abbreviations: *BMI* body mass index, *ABG* arterial blood gas, *NIV* non invasive ventilation, *LVEF* left ventricular ejection fraction

Seven nights after the NIV was discontinued, polygraphy of the weaning period showed a high mean AHI (AHI = 53.5/h ±26.9) and the mean RT < 90% was 21.8% (24.2) (Table 2).

**Table 2:** Wash out period: pulmonary function tests and polygraphy

PFT and Polygraphy (during withdrawal)	*n* = 15
FEV1/FVC (%)	79.5 ± 7.4
FEV1 (%)	69.2 ± 16.4
FVC (%)	70.2 ± 17.3
AHI (n/h)	53.5 ± 26.9
RT < 90% (%)	21.8 ± 24.2
ODI ≥ 4% (n/h)	42.1 ± 39.5

Data are expressed in mean ± standard deviation

Definition of abbreviations: *PFT* pulmonary function test, *FEV1* forced expiratory volume in 1 s, *FVC* forced vital capacity, *AHI* apnea hypopnea index, *RT < 90%* percentage of recording time below 90% saturation, *ODI* oxygen desaturation index

Regarding settings, a﻿﻿ll﻿ NIV were in spontaneous timed (ST) mode, mean EPAP was 7.7 ± 1.8 cm H2O, mean IPAP was 18.3 ± 3 cm H2O, mean back-up RR was 13.9 ± 1.8 cycles/min. Two patients received additional oxygen therapy, flow rates were not modified after switching to CPAP. (Table 3).

**Table 3:** NIV and CPAP settings

NIV and CPAP settings	*n* = 15
NIV mode	ST
NIV EPAP (cm H2O)	7.7 ± 1.8
NIV IPAP (cm H2O)	18.3 ± 3
NIV RR (n/min)	13.9 ± 1.8
NIV VT (ml)	508.3 ± 264
CPAP EPAP (cm H2O)	9.8 ± 1.4
Oronasal mask	10 (66.7%)
Nasal mask	3 (20%)
Nasal pillows	2 (13.3%)

Data are expressed in mean ± standard deviation and n (%)

Definition of abbreviations: *NIV* non invasive ventilation, *CPAP* continuous positive airway pressure, *EPAP* expiratory positive airway pressure, *IPAP* inspiratory positive airway pressure *RR* respiratory rate, *VT* tidal volume

### Effect of the switch to CPAP on the measured data

**Fig. 3:**
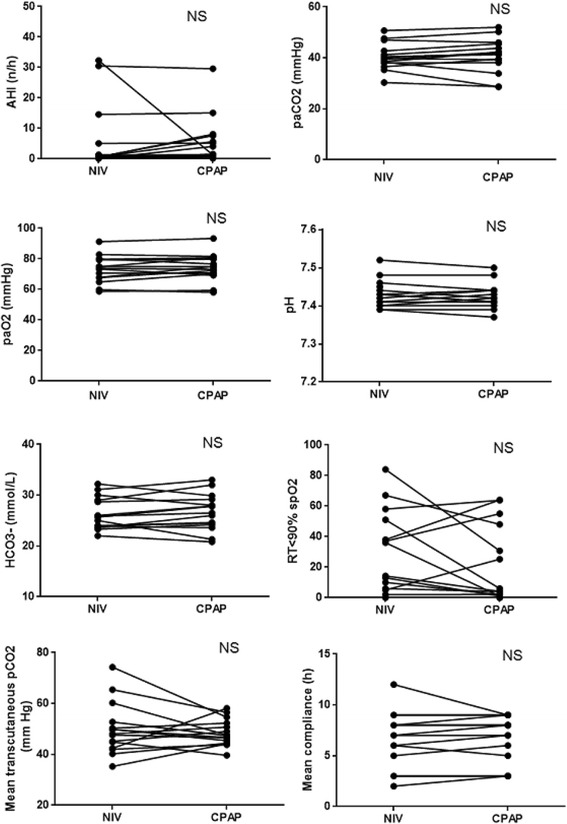
Measurement of mean AHI; diurnal and nocturnal alveolar gas exchange parameters and mean compliance, in 15 patients, with NIV and one month after the switch to CPAP. Definition of abbreviations: NIV: Non Invasive Ventilation; CPAP: continuous positive airway pressure; AHI: Apnea Hypopnea Index; RT < 90% spO2: percentage of recording time below 90% saturation; NS: no significant difference *p* > 0.05

There were no significant differences, after the switch to CPAP, in mean AHI, diurnal and nocturnal alveolar gas exchanges (diurnal ABG, nocturnal oximetry and transcutaneous capnometry), median leaks and compliance (Table 4, Fig. 3).

**Table 4:** Measured data with NIV and one month after the switch to CPAP

Measured data	NIV	CPAP	*P* value
Pa CO2 (mm Hg)	40.4 ± 5.1	40.9 ± 6.7	0.323
Pa O2 (mm Hg)	73.3 ± 8.8	74.4 ± 8.8	0.235
pH	7.43 ± 0.04	7.43 ± 0.03	0.508
HCO3‾ (mmol/L)	26.3 ± 3.14	26.6 ± 3.6	0.511
RT < 90% (%)	28.1 ± 27.2	20.5 ± 25.2	0.218
ODI ≥ 4% (n/h)	11.92 ± 17.31	10 ± 9.41	0.285
Transcutaneous pCO2 (mm Hg)	49.9 ± 10.12	48.4 ± 5.14	0.534
AHI (n/h)	5.99 ± 10.96	5.49 ± 7.84	0.334
Leaks (L/sec)	3.8 ± 10.09	2.9 ± 6.1	0.844
Compliance (h)	6.73 ± 2.66	6.8 ± 2.27	0.766

Data are expressed in mean (standard deviation)

Definition of abbreviation: *NIV* non invasive ventilation, *CPAP* continuous positive airway pressure, *RT < 90%* percentage of recording time below 90% saturation, *ODI* oxygen desaturation index, *AHI* apnea hypopnea index

**Fig. 4:**
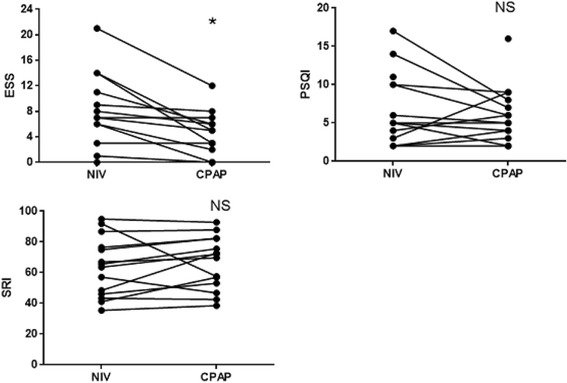
Changes in ESS, PSQI and SRI scores one month after the switch to CPAP. Definition of abbreviations: NIV: Non Invasive Ventilation; CPAP: Continuous Positive Airway Pressure; ESS: Epworth Sleepiness Scale, PSQI: Pittsburgh Sleep Quality Index; SRI: Severe Respiratory Insufficiency questionnaire; NS: no significant difference; *: significant difference *p* < 0.05

Regarding ESS, SRI and PSQI scores, the only significant difference was in the ESS, which was lower after the switch to CPAP (8.21 ± 5.54 vs 4.29 ± 3.43) (*p* = 0.004). There were no significant differences in the SRI and all its subscales and in the PSQI and all its components between before and after the switch to CPAP. (Table 5, Fig. 4).

**Table 5:** ESS, PSQI and its components, SRI and its subscales scores with NIV and one month after the switch to CPAP

Scores	NIV	CPAP	*P* value
ESS	8.21 ± 5.54	4.29 ± 3.43	0.004*
PSQI	6.86 ± 4.77	6.14 ± 3.65	0.311
Componant 1	1.07 ± 0.92	0.86 ± 0.53	0.500
Componant 2	0.79 ± 1.37	1.29 ± 1.27	0.250
Componant 3	0.93 ± 0.83	0.93 ± 1.07	1
Componant 4	0.5 ± 0.85	0.57 ± 0.85	0.531
Componant 5	1.29 ± 0.61	1.21 ± 0.43	1
Componant 6	1.21 ± 1.48	0.43 ± 1.09	0.125
Componant 7	1 ± 1.18	0.86 ± 1.03	0.766
SRI	62.4 ± 19.46	66.5 ± 17.32	0.153
SRI RC	70 ± 21.61	71.87 ± 21.58	0.529
SRI PF	48.61 ± 28.33	46.13 ± 29.17	0.404
SRI AS	54.83 ± 28.80	64.54 ± 22.04	0.135
SRI SF	70.83 ± 21.30	75 ± 19.81	0.682
SRI ANX	69.67 ± 16.74	70.14 ± 18.02	0.968
SRI WB	61.31 ± 28.63	73.21 ± 21.89	0.240
SRI SR	61.54 ± 23.52	64.38 ± 22.25	0.675

Data are expressed in mean (standard deviation)

Definition of abbreviations: *NIV* non invasive ventilation, *CPAP* continuous positive airway pressure, *ESS* epworth sleepiness scale, *PSQI* Pittsburgh sleep quality index, *SRI* severe respiratory insufficiency questionnaire, *RC* respiratory complaints, *PF* physical functioning, *AS* attendant symptoms, *SF* social functioning, *ANX* anxieties, *WB* well being, *SR* social relationships

*means significance with *p* value < 0.05

At the end of the trial, through a self-administered questionnaire, the patients’ preference for one mode of ventilation was determined. Twelve patients (80%) reported that they preferred CPAP over NIV and wanted to continue their treatment with CPAP. Two patients (13%) had no preference. Only one patient wanted to discontinue CPAP and go back to NIV, despite the fact that there were no objective criteria of deterioration during CPAP treatment.

### Safety

None of the patients presented with clinical deterioration after more than a month of CPAP and needed to be switched back to NIV. Two patients were admitted in the intensive care unit during the trial, due to causes unrelated to the mode of ventilation. One presented with acute renal failure complicated by symptomatic hyperkalemia and the other with left heart failure secondary to non-compliance to diuretics.

### Cost

When taking into account French social security reimbursement rate, NIV cost was 63.16€ per week and CPAP cost was 18€ per week. This switch may allow an economy of 2348€ per year per patient.

### NIV failure

When analyzing the characteristics of the patients failing NIV, according to Salord’s criteria: more than 30% of recording time spent below 90% saturation or paCO2 > 45 mm Hg; BMI was the only factor associated with NIV failure (*p* = 0.014) (Table 6).

**Table 6:** NIV failure predictor factors

Clinical characteristics	NIV failure (*n* = 8)	NIV success (*n* = 7)	*P* values
Age (years)	67.12 ± 8.64	70.71 ± 14.04	0.555
BMI (kg/m^2^)	46.54 ± 4.84	36.2 ± 5.07	0.014*
Neck circumference (cm)	45.12 ± 5.64	41.93 ± 1.97	0.179
Waist/Hip ratio	0.94 ± 0.08	0.97 ± 0.09	0.448
paCO2 at diagnosis (mm Hg)	57.12 ± 8.56	58.29 ± 6.97	0.780
Cardiovascular comorbidities	7 (87.5%)	6 (85.7%)	1
FVC (%)	68.07 ± 19.46	70.49 ± 13.48	0.788
AHI (n/h)	63.09 ± 28.53	42.54 21.76	0.145
RT < 90% (%)	26.45 ± 28.55	16.54 ± 18.89	0.450
Diagnosis during an exacerbation	6 (75%)	6 (85.7%)	0.605

Data are expressed in n (%); mean (standard deviation)

Definition of abbreviations: *NIV* non invasive ventilation, *BMI* body mass index, *FVC* forced vital capacity, *AHI* apnea hypopnea index, *RT < 90%* % of recording time below 90% saturation

## Discussion

This prospective open trial, testing the feasibility of a protocol switching NIV to CPAP in stable patients in real life conditions, suggests the possibility of this switch in patients suffering from OHS with an AHI ≥ 15/h and without pulmonary obstructive disease. In these conditions, we reported no significant treatment effect differences between before and after the switch to CPAP in diurnal and nocturnal alveolar gas exchange, mean AHI, mean compliance, quality of sleep and quality of life. In addition, 80% of patients preferred CPAP over NIV.

The pathophysiology of OHS is complex and multifactorial. Eventually leading to chronic, diurnal hypercapnia resulting from an imbalance between apnea and inter-apnea periods and deficiency of the compensatory mechanisms to unload the carbon dioxide in excess [17, 18]. This suggests the need to determine different phenotypes of OHS, and therefore adapt therapeutic intervention according to the phenotype. Therefore we have chosen in this trial, to exclude from the study, patients with an AHI < 15/h, speculating that CPAP mechanism to treat hypoventilation was mostly due to the correction of apneas and hypopneas. Indeed, if CPAP is not intrinsically the most efficient treatment of hypoventilation, it is still effective on most of the pathophysiological mechanisms leading to OHS by decreasing upper airways resistance, increasing central response to hypercapnia and hypoxemia [19, 20], increasing lung volumes, treating atelectasis, decreasing intrinsic PEEP [21]. This is the reason why CPAP is efficient in most patients with OHS associated with OSA.

Two prospective randomized trials have compared the efficacy of CPAP and NIV in patients with OHS [5, 11]. These two prospective studies corroborate the fact that patients with OHS and concomitant OSA can be treated with CPAP. Piper and colleagues did not find a significant difference, at three months, in daytime paCO2 between the two groups NIV and CPAP [11]. However in this study, nine of the 45 patients initially selected were excluded from the trial, due to persisting nocturnal hypoxemia or nocturnal hypercapnia on the first night of CPAP titration. Treating hypoventilation in some phenotypes may take up to a few weeks [22], excluding patients after one night of CPAP titration is debatable. Masa and colleagues confirmed the absence of a significant difference between NIV and CPAP, at two months, in diurnal paCO2 [5]. However, only NIV and not CPAP significantly improved paCO2 compared to the control group. An improvement was only noted for patients using CPAP more than 4 h per night. Moreover, the authors found a significant difference, favoring NIV, on the 6 min’ walk test and respiratory function tests.

The heterogeneity in responses to CPAP, led some studies to try to define phenotypes of OHS and predictor factors of CPAP failure. Therefore, patients exhibiting a higher BMI, a higher percentage of recording time below 90% saturation, a lower AHI, or a decreased forced vital capacity could be at higher risk of CPAP failure, even though these predictor factors are not constantly found in all studies [8–10] . In our study, we found that only BMI had a significant association with CPAP or NIV failure (Table 6). When using the two criteria defining failure in Salord and colleagues study [10] (more than 30% of recording time spent below 90% saturation or paCO2 > 45 mm Hg), in our study 8/15 patients would be categorized as NIV failure and 7/15 as CPAP failure. Interestingly, the seven patients failing CPAP were the same patients who were failing NIV before the switch. We do not think this failure can be attributed to NIV or CPAP settings in our study. Mean EPAP was 8 cm H2O, mean IPAP was 18 cm H2O, back-up RR was 14 cycles/min, all NIV were in ST mode. Concerning CPAP, mean EPAP was 9.8 cm H2O. These pressures are concordant with previous trials [5, 11].

Our study has some limitations. First of all it can be argued that the sample size in our study (15 patients) could be insufficient to detect a significant difference after the switch to CPAP, and that this absence of difference could be due to a lack of power. This study does not have the pretention to assert the absence of difference between NIV and CPAP at a larger scale, but describes a feasible protocol for most sleep clinic, to switch stable patients with OHS from NIV to CPAP. As suggested by our preliminary results, for some patients, the switch of NIV to CPAP can be deleterious emphasizing the need for a careful monitoring. A second limitation is that the results of our study can only be extended to patients with OHS and concomitant OSA with AHI ≥ 15/h, without obstructive pulmonary diseases, and cannot be used when treating patients in an acute state or with mild OSA. These drastic selection criteria aimed at targeting CPAP responders’ phenotypes and the limited number of patients included in the study could partly explain the stable diurnal capnia after the switch to CPAP in all of our patients. The treatment of patients with OHS with pure hypoventilation and without OSA, is seldom discussed in scientific literature, and these patients still benefit from NIV in most teams [23]. For ethical reasons, we decided not to switch to CPAP, patients with mild OSA (AHI < 15/h), to not deprive these patients with previous acute exacerbation from an effective therapy.

To eliminate NIV persistent effect, we introduced in the protocol, a withdrawal from NIV for seven nights before starting CPAP, and reevaluated patients more than a month after starting CPAP. It is possible that not all persistent effects attributable to NIV were eliminated by this withdrawal [24]. An ideal protocol would have been to randomize patients into three groups: one group pursuing NIV, one group switching to CPAP and one group stopping ventilation; but this protocol did not seem ethical for patients withdrawn from ventilation, considering that a higher mortality rate has been reported in untreated patients with OHS [4, 6, 7]. In our study, 12 out of 15 patients were diagnosed with OHS and started on NIV, following an episode of exacerbation. This raises the question as to whether the diagnosis of OHS was made in excess in patients with normocapnic OSA undergoing an exacerbation. This assertion is however contradicted by the fact that nine out of the 12 patients diagnosed with OHS following an exacerbation, had high bicarbonates level (HCO3- > 28 mmol/l), in favor of chronic hypercapnia [25]. When measuring the AHI, we used the built-in software available in the NIV and the CPAP. Although the accuracy of these systems is debatable, recent studies are in favor of a good reliability of these built-in softwares [26].

## Conclusion

In conclusion, this preliminary study showed in real life conditions, the possibility of a systematic switch of NIV to CPAP in most stable patients with OHS and a concomitant OSA, with similar efficacy on diurnal and nocturnal alveolar gas exchange, quality of life and quality of sleep. These results need to be confirmed by a larger trial that could also evaluate the clinical consequences of this switch in the longer term, especially cardio-vascular consequences.

## Abbreviations

AASM: American Academy of Sleep Medicine
ABG: Arterial blood gas
AHI: Apnea hypopnea index
BMI: Body mass index
CPAP: Continuous positive airway pressure
EPAP: Expiratory positive airway pressure
EPS: Epworth sleepiness scale
FEV1: Forced expiratory volume in 1 second
FVC: Forced vital capacity
HCO3-: Bicarbonates
IPAP: Inspiratory positive airway pressure
NIV: Noninvasive ventilation
ODI: Oxygen desaturation index
OHS: Obesity hypoventilation syndrome
OSA: Obstructive sleep apnea
PaCO2: Partial pressure of arterial carbon dioxide
PaO2: Partial pressure of arterial oxygen
PFT: Pulmonary function tests
PSQI: Pittsburgh sleep quality index
PtCO2: Transcutaneous measurement of carbon dioxide
RR: Respiratory rate
RT < 90%: percentage of recording time with SaO2 < 90%
SaO2: Oxygen saturation
SD: Standard deviation
SRI: Severe respiratory insufficiency questionnaire
ST: Spontaneous timed mode
